# Pharmacological and bioactive properties of *Artemisia argyi* H. Lév. & Vaniot essential oil: a review

**DOI:** 10.3389/fphar.2025.1664658

**Published:** 2026-01-07

**Authors:** Qing Gao, Yanqin Lu

**Affiliations:** 1 School of Pharmacy and Institute of Pharmaceutical Research, Shandong First Medical University and Shandong Academy of Medical Sciences, Ji’nan, Shandong, China; 2 Key Laboratory for Biotech-Drugs of National Health Commission, Key Laboratory for Rare and Uncommon Diseases of Shandong Province, Biomedical Sciences College and Shandong Medicinal Biotechnology Centre, Shandong First Medical University and Shandong Academy of Medical Sciences, Ji’nan, China; 3 Department of Endocrinology and Metabology, The First Affiliated Hospital of Shandong First Medical University and Shandong Provincial Qianfoshan Hospital, Ji’nan, China

**Keywords:** anti-cancer cell proliferation, anti-inflammation, antioxidant, anti-pathogenic microorganism, *Artemisia argyi*, essential oil, immunomodulation

## Abstract

*Artemisia argyi* H. Lév. & Vaniot Essential Oil (AAEO) is a volatile oil extracted from the leaves and stems of *Artemisia argyi*. It contains bioactive plant metabolites such as terpenes, aldehydes, ketones, alcohols, and esters. AAEO has antioxidant properties that help remove harmful free radicals and protect cells from damage. It also exerts anti-inflammatory effects by inhibiting the activation of NLRP3 inflammasome (NLR family pyrin domain-containing 3 inflammasome) and the phosphorylation of NF-κB p65, thereby reducing the levels of pro-inflammatory metabolites such as IL-1β, IL-6, and TNF-α. Additionally, AAEO has antimicrobial effects against bacteria, fungi, and viruses. In cancer cell studies, it can slow down the growth and migration of certain cancer cells by inducing apoptosis or inhibiting proliferation. AAEO shows potential in several areas, its insecticidal activity suggests it could be used as a natural mosquito repellent. There are three main challenges with AAEO. First, its chemical composition can vary depending on where it is grown, which affects its stability and effectiveness. Second, most of the evidence for its benefits comes from studies on cells or animals, with few human clinical trials. Third, AAEO has low bioavailability, meaning it is not easily absorbed by the body. Future research should focus on creating unified quality standards for AAEO, conducting large-scale clinical trials to confirm its safety and effectiveness, and developing advanced delivery systems to enhance its absorption and stability.

## Graphical Abstract

**Figure:**
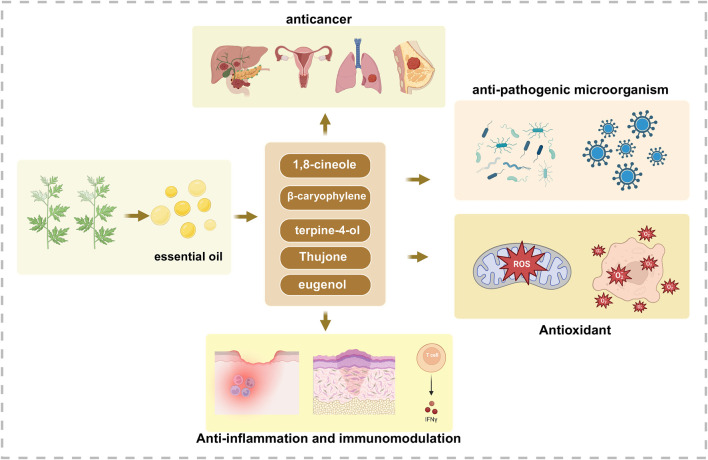

## 1 Introduction

*Artemisia argyi* H. Lév. & Vaniot, also known as xiang ai or jia ai in Chinese, is a perennial plant belonging to the Artemisia genus in the Asteraceae family. As an important traditional botanical drug, it has been used for over 2,000 years in Eastern countries, with a long history of medicinal application in China (Liang et al., 2020). *Artemisia argyi* H. Lév. & Vaniot is a traditional botanical drug that has been used for over 2,000 years in Eastern countries. *Artemisia argyi* H. Lév. & Vaniot Essential Oil (AAEO), as the main active fraction of this botanical drug, is a volatile oil extracted from its leaves and stems. These plants have a strong aroma, the stems are brown or grayish-yellow with a few short branches, and the leaves are thick, papery, and covered with short grayish-white hairs (Nie et al., 2019). Mainly distributed in eastern Asia, *A. argyi* grows in almost all parts of China. It shows strong adaptability, and grows well even in the extremely dry and cold parts of China. Ancient books divided *A. argyi* into bei ai, qi ai, and hai ai (Jiang et al., 2019). The *A. argyi* variety native to Qichun County, Hubei Province, is the earliest developed and most widely used variety of this botanical drug in China (Jiang et al., 2021). The whole plant of *A. argyi* can be used as a source of this botanical drug, exhibiting functions of removing dampness, dispelling cold, promoting hemostasis, relieving inflammation, allergies, asthma, and coughs, and preventing miscarriage (Liu et al., 2017; Liu et al., 2018). Several historical records describe this plant and its uses. Tao Hongjing, a noted physician and naturalist in the Southern Dynasty, noted in Records of Famous Physicians that: “Ai leaves taste bitter and they are non-toxic. They are primarily used for moxibustion to treat various illnesses. They can also be decocted into a medicinal soup to regulate yin qi, promote muscle growth, expel wind and cold, and help individuals conceive.” Zhang Zhongjing’s Golden Cabinet Prescriptions, written during the Han Dynasty, noted: “For women experiencing continuous uterine bleeding, prolonged bleeding after miscarriage, or bleeding during pregnancy, if abdominal pain occurs during pregnancy due to uterine obstruction, the jiao ai decoction is prescribed as the treatment.” The Compendium of Materia Medica written by Li Shizhen during the Ming Dynasty records: “Moxibustion, doctors used for all diseases, so it is called moxibustion herb.” Beyond its deep roots in traditional Chinese medicine, *A. argyi* is growing world-wide recognition as can be seen from its growing international trade and cultural convergence adoptions. One registered pilot observational study was registered at the University Hospital Freiburg (Clinical Trials.gov Identifier: NCT01126931) in order to evaluate the beneficial effects of A. argyi supplementation on appetite in patients with different chronic progressive diseases with poor appetite as part of their respective diseases or conditions. These diseases or conditions included cancer, autoimmune diseases (including Crohn’s disease and IgA nephropathy), depression, and geriatric frailty. This clinical research was one example of how to apply a scientific method to recognize the value of *A. argyi* in a European medical context.

AAEO is the volatile oil isolated from the leaves and stems of *A. argyi* (Qian et al., 2022). The extraction rate of essential oil is higher from *A. argyi* leaves than from stems (Xu et al., 2016), and thus the leaves are most commonly used to extract AAEO in commercial production. AAEO is yellow or light green in color, with a strong fragrance (Wang M. D., et al., 2022). It has a wide range of beneficial pharmacological properties such as antibacterial, antifungal, antiviral, anti-inflammatory, antioxidant, and anti-cancer cell activities (e.g., inhibiting cancer cell proliferation and inducing apoptosis) (Abiri et al., 2018; Han et al., 2017; Lee et al., 2020; Cao et al., 2018). The composition of AAEO is relatively complex, and includes terpenoids, aldehydes (ketones), alcohols (phenols), acids (esters), and alkanes (olefins) (Wu et al., 2022). To date, more than 200 plant metabolites have been identified in AAEO, including terpenes (e.g., 1,8-cineole, β-pinene, D-germacrene), ketones (e.g., camphor, thujone), alcohols (e.g., borneol), and esters (e.g., ethyl hexadecanoate). A previous study analyzed the volatile oils of several different *A. argyi* materials by gas chromatography–mass spectrometry, and the chromatographic peaks were qualitatively identified through comparison with data in spectral databases and data reported in the literature (Chen et al., 2021). A total of 39 plant metabolites were identified, among which only 13 were found in all the materials: 1,8-cineole, agaritol, trans-terpinol, D-camphor, 4-terpenol, α-terpinol, myrtenol, carvol, L-carvone, β-caryophyllene, dactylodene D, caryophyllene oxide, and 2, 4-di-tert-butylphenol. Among these plant metabolites, agaritol, α-terpinol, terpenes (including caryophyllene, etc.), and camphor derivatives (including D-camphor, etc.) are aromatic substances with strong odors and most of them also have well-defined pharmacological activities (Chen et al., 2021). The remaining compounds also show potential anti-inflammatory, antiviral, or anticancer properties, as detailed below: 1,8-Cineole possesses anti-inflammatory, antiviral, and anticancer activities. It can inhibit the secretion of inflammatory factors, directly inactivate infectious bronchitis virus (IBV), and exhibit *in vitro* antiproliferative effects on colorectal cancer Caco-2 cells (Pries et al., 2023). α-Terpineol can modulate inflammatory signaling pathways to alleviate tissue damage and exhibits certain antiviral activities, making it an important component of essential oils for combating infections (Zheng et al., 2025). D-Camphor can improve local circulation, alleviate inflammatory swelling and pain, and possesses potential antiviral activity. It is commonly used topically for the treatment of skin inflammation (Kovaleva et al., 2018). Carvacrol inhibits the release of IL-6 and TNF-α by activating the autophagy pathway. Its anti-inflammatory effects are associated with the regulation of mitochondrial function. Moreover, carvacrol can inhibit tumor cell proliferation at low doses with minimal toxicity (Lu et al., 2025). Myrtanol, due to the anti-inflammatory and antiviral properties of its congener myrtenol, is speculated to possess similar immunomodulatory and phytogenic anti-infectious potential (Zhang N. et al., 2023). Given the characteristics of α-terpineol, it is hypothesized that 4-terpineol and trans-terpineol possess anti-inflammatory and antibacterial activities, and can support the soothing effects of essential oils (Zhu et al., 2025). Agarose, L-carvone, dodecene, and 2,4-di-tert-butylphenol, although with limited reports on their direct pharmacological effects, may enhance the efficacy of other active compounds through synergistic interactions (Zhang Z. L. et al., 2023). This review focuses on the latest progress in research on the pharmacological activity of AAEO and provides support for its scientific and rational utilization. The taxonomic validity of *A. argyi* H. Lév. & Vaniot has been verified via Plants of the World Online (http://www.plantsoftheworldonline.org), and it is recognized as an accepted species in the family Asteraceae, genus Artemisia, with no synonym disputes or taxonomic ambiguities. To meet the ethnopharmacological research standards, this study summarizes the deficiencies and improvements of current research, based on the Four Pillars of Ethnopharmacology (Table 1).

**TABLE 1:** Four pillars of ethnopharmacology compliance.

Four pillars category	Current gaps	Simplified improvements	Placement in manuscript	Guideline alignment
1. Cultural Context	Lack of ethnic/regional use scenarios; no clarity on variety-specific traditional uses	1. Add: *A. argyi* use in Han (moxibustion), Miao (fumigation), Korean-Chinese (external wash) medicine (Jiang et al., 2019)	Intro (after Compendium of Materia Medica cite); Intro (after Qichun variety desc.).	Clarify ethnic origin & variety-specific traditional uses
		2. Add: Qichun Ai (miscarriage preventi on) vs. Cang-ai (headache relief) (Jiang et al., 2021)		
2. Botanical Authentication	Scattered auth. info for related plants; no voucher specimen/POWO IDs	1. Update “2.1 Core Plant Table”: Add Voucher No. (e.g., *A. argyi*: SDFMU-AA-202205) & POWO ID (e.g., 50325242)	Revised “2.1 Core Plant Table” (new columns)	Ensure traceability via voucher IDs & database credentials
		2. Add validation date (2024-10)		
3. Methodological Rigor	Unclear AAEO extraction params; no quality controls/animal ethics No.	1. Add AAEO extraction: steam distillation (3h, 1:10 ratio, 2.1%–2.5% yield) (Xu et al., 2016)	Intro (after Xu et al., 2016); New “2.2 AAEO QC”; Pharmacol. sections (pre-animal desc.).	Specify protocols, QC, & ethics for reproducibility
		2. Add: 1,8-cineole ≥30%, camphor ≤15% (Chin. Pharmacopoeia, 2020)		
		3. Add ethics No.: SYXK-2023-0012		
4. Traditional Use-Pharmacology Link	No direct link between traditional uses & modern pharmacology; no toxicity-dosage match	1. Add link: Traditional anti-inflammation → JAK/STATs inhibition (Chen et al., 2017); hemostasis → enhanced platelet aggregation (Wang et al., 2017)	New “3.1 Trad.-Modern Link”; Conclusion (safety para.).	Establish causal links between traditional use & modern data
		2. Add: Traditional dose (5–10g/moxibustion) matches 2% AAEO safety (Luo et al., 2022)		

## 2 Literature search strategy

A systematic literature search was conducted in PubMed, Web of Science, CNKI and Wanfang from January 1981 to October 2025. The following English and Chinese terms were combined: (“*A. argyi* essential oil” OR “AAEO”) AND (“pharmacological activity” OR “antioxidant” OR “anti-inflammatory” OR “inhibition of cancer cell proliferation and induction of cancer cell apoptosis” OR “antimicrobial”). After removal of duplicates, titles and abstracts were screened for relevance and then the full texts were evaluated against pre-defined eligibility criteria. To make sure the taxonomic validity of all plant species in the core pharmacological experiment of included studies, we summarized the taxonomic information and validation results of key plants (the core research object *A. argyi* and its related reference species, Table 2).

**TABLE 2:** Anticancer effects of AAEO and its component active plant metabolites.

Active substance	Chemical structure	Types of cancer cells	Mechanism of action	References
AAEO	-	HCC	Downregulate the expression of DEEDC1 gene and block the Wnt/β - catenin pathway	Li B. et al. (2021)
AAEO		HPAC	Promote the imbalance of iron ions, destroy the γ - glutamyl cycle and increase the content of polyunsaturated fatty acids	Zhang N. et al. (2023)
AAEO		B16F10	Downregulate tyrosinase activity or alleviate oxidative stress	Kim and Hong (2024)
β - Caryophyllene and β - Caryophyllene Oxide	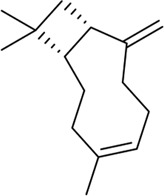	MCF-7, PC-3	Induction of MAPK activation and inhibition of PI3K/AKT/mTOR/S6K1 signaling pathway	Fidyt et al. (2016)
1,8-Cineole	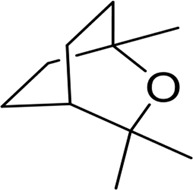	HaCaT	Reducing COX-2 expression to inhibit skin cancer growth and epidermal thickening induced by medium wave ultraviolet radiation (290–320 nm)	Lee, et al. (2017)
1,8-Cineole		A549	Inhibiting A549 cell growth and migration through cell cycle arrest, and making cells sensitive to simvastatin	Rodenak-Kladniew, et al. (2020a)
1,8-Cineole		HepG2	Promote G0/G1 cell cycle arrest in HepG2 cells, induce aging through oxidative stress, and make cells sensitive to anti-aging drugs	Rodenak-Kladniew et al. (2020b)
thujone	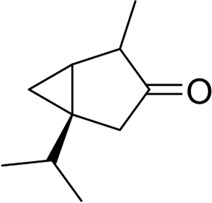	GBM	Reduce cell viability, inhibit proliferation, promote apoptosis, and resist angiogenesis	Torres et al. (2016)
thujone		SiHa	Acting on mTOR, PI3K, AKT1, Bcl-2, and MDM2 targets, exerting anti-cancer effects	Pal et al. (2022)
camphene	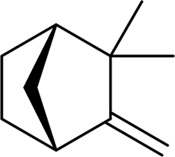	B16F10-Nex2	Inducing significant condensation of endoplasmic reticulum in melanoma cells and activating the body’s immune system	Girola et al. (2015)

## 3 Pharmacological effects of AAEO

The broad pharmacological spectrum of AAEO is inherently attributed to the physicochemical characteristics of typical terpenoid components in AAEO, including 1,8-cineole, camphor, and borneol. These compounds exhibit low molecular weight and high lipophilicity and diffuse into biological membranes, including the plasma membrane and the blood-brain barrier, rapidly through passive diffusion. This mode of cellular uptake does not involve specific channels or receptors; therefore, low-molecular-weight and highly lipophilic compounds have rapid intracellular access and can modulate various targets, including enzymes, signaling proteins, and organelles, which are involved in activities discussed in the following sections (Huang et al., 2025).

### 3.1 Antioxidant effect

Many studies have shown that the occurrence of aging and various diseases is closely related to the accumulation of free radicals (Mao et al., 2019). Excess free radicals induce oxidative stress, which damages DNA, proteins, and lipids to impair cellular structure. This ultimately contributes to diseases including cancer, atherosclerosis, Parkinson’s disease, stroke, diabetes, rheumatoid arthritis, and Alzheimer’s disease (Fang et al., 2002; Lobo et al., 2010; Liu et al., 2011; Wong et al., 2012). Antioxidants interfere with the oxidation process by scavenging free radicals and reactive oxygen species (ROS) and/or chelating oxidants with various metal ions (Sárdy, 2009; Wlaschek et al., 1995). Thus, antioxidants play a crucial role in maintaining cell health and treating free radical-related diseases.

Previous studies have systematically evaluated the antioxidant activity of AAEO using a variety of methods, including DPPH and ABTS radical scavenging, reduction of oxidized substrates, and metal ion chelation (Huang et al., 2012; Oyaizu, 1986; Wlaschek et al., 2001; Zhang et al., 2020). Specifically, AAEO exhibited concentration-dependent DPPH scavenging activity with an IC50 of 0.31 μL/mL (Zhang et al., 2020), and at concentrations of 0.045, 0.225, and 0.450 mg/mL, its ABTS scavenging rates reached 61.49% ± 1.12%, 75.7% ± 1.16%, and 91.41% ± 0.57% (p < 0.001 vs. positive control), respectively (Huang et al., 2012). For instance, one study reported an IC50 value of 7.84 ± 1.70 μg/mL for AAEO derived from needles, whereas another study showed that two samples of AAEO obtained by different methods from the twigs with cones had EC50 values of 32.0 ± 0.03 mg/mL and 29.93 ± 0.08 mg/mL, respectively (Vukić et al., 2025). These differences can be ascribed to the chemical composition of the essential oils tested, which is influenced by the plant material used for essential oil extraction, the method employed for essential oil isolation, and the method utilized to determine DPPH neutralization (Vukić et al., 2025).

However, the strong antioxidant activity observed *in vitro* cannot be fully replicated in in vivo models, and the underlying mechanisms differ significantly. For example, studies have shown that oral administration of AAEO (100 mg/kg/day) in aging mice only increased hepatic GPx activity by 46.5% and reduced MDA levels by 26.8% (Haridy et al., 2020). This is far lower than the 91.41% ABTS scavenging rate observed *in vitro* at 0.45 mg/mL (Peng et al., 2024). This discrepancy is attributed to the rapid *in vivo* metabolism of AAEO: studies have found that 1,8-cineole (the main antioxidant component of AAEO) has a plasma half-life of only 1.2 h in rats, with 85% metabolized to inactive 2-hydroxy-1,8-cineole within 4 h (Ahmadi et al., 2024). Additionally, recent research has demonstrated that AAEO alleviates ethanol-induced liver oxidative stress by regulating intestinal flora (increasing Bifidobacterium abundance) and upregulating IL-10— a mechanism not observed in in vitro HepG2 cell models treated with H2O2 (Huang et al., 2024). These findings highlight that *in vitro* experiments only reflect direct radical-scavenging capacity, while *in vivo* effects depend on host metabolism and microecology, indicating a critical need for translational studies to validate antioxidant efficacy across scales.

**FIGURE 1:**
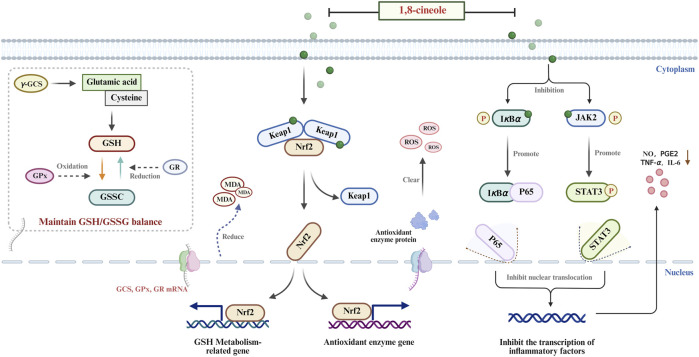
Antioxidant mechanism of AAEO. AAEO exerts antioxidant effects through following mechanisms: balancing GSH/GSSG by regulating critical enzymes, activating the Nrf2 pathway and regulating the JAK-STAT and the NF-κB pathways.

Hydroxyl substituted aromatic rings in phenolic plant metabolites are able to form resonantly stable phenoxy groups to quench free radicals (Šiler et al., 2014). The more hydroxyls groups in the structure of phenolic acid plant metabolites, the stronger their antioxidant capacity (Xia et al., 2019). Moreover, because the antioxidant capacity is closely related to the ability of the molecule to provide protons, the hydroxyl groups in flavonoids can exert their activity only if they form a stable free radical structure (Heim et al., 2002). The antioxidant mechanism of 1, 8-cineole, a component of AAEO, is shown in Figure 1.

Oxidative stress refers to the imbalance between the generation of ROS and the endogenous antioxidant defense system. Under normal physiological conditions, cells produce small amounts of ROS, which are involved in cell signaling and are able to be rapidly removed by the antioxidant defense system. However, under pathophysiological conditions, the excessive production of ROS exceeds the regulatory capacity of the antioxidant defense system, leading to cell damage and even death (Van Der Pol et al., 2019).

Enzymes that govern glutathione (GSH) turnover are pivotal for maintaining intracellular redox homeostasis and antioxidant competence. As a pivotal antioxidant enzyme, Glutathione peroxidase (GPx) utilizes reduced GSH as a substrate to detoxify harmful hydrogen peroxide (H2O2) and lipid hydroperoxides, converting them into innocuous water or lipid alcohols while concurrently oxidizing GSH to glutathione disulfide (GSSG), thereby preventing the propagation of oxidative damage. According to Zheng et al. (2020), *A. argyi* essential oil (AAEO) significantly elevates GPx activity in murine heart, liver, and kidney. At an oral dose of 100 mg kg^-1^ day^-1^, GPx activity increased by 46.5% in the liver, 78.3% in the heart, and 80.4% in the kidney compared with the aging-model group. Concomitantly, hepatic GSH content rose by 54.0% and malondialdehyde levels decreased by 26.8%, accompanied by marked histopathological improvement (Zheng et al., 2020). These findings indicate that AAEO reinforces the glutathione-dependent peroxide-scavenging system and mitigates oxidative stress-related injuries such as liver damage and organ aging. Glutathione reductase (GR) catalyzes the NADPH-dependent reduction of oxidized glutathione (GSSG) to its reduced form (GSH), thereby functioning as the principal “recycling enzyme” that sustains intracellular GSH homeostasis and prevents GSH depletion during continuous antioxidant reactions. Zhang et al. (2022) reported that a 7-day oral administration of AAEO at 100 mg kg^−1^ increased GR activity by 26.7% and elevated the GSH/GSSG ratio to 1.45-fold that of the control, thereby accelerating the GSSG→GSH cycle and reinforcing the organism’s resistance to oxidative damage. Glutathione-S-transferase (GST) are phase II detoxifying enzymes that catalyze the nucleophilic addition of the thiol group (-SH) of glutathione (GSH) to electrophilic xenobiotics, drug metabolites, or lipid peroxidation products (Hayes et al., 2005). This conjugation generates water-soluble GSH adducts that are readily excreted in urine, thereby minimizing the accumulation of toxic plant metabolites within the body. Although direct measurement of glutathione S-transferase (GST) activity has not been reported in current *A. argyi* essential oil (AAEO) studies, the documented global activation of the glutathione system—increased GSH availability and upregulation of GPx and GR—suggests that AAEO may indirectly enhance GST function (Cui et al., 2024). Such an effect would support hepatic detoxification and contribute to the amelioration of toxicant-induced liver injury (e.g., ethanol- or bisphenol A-mediated hepatotoxicity). γ-Glutamyl-cysteine synthetase (γ-GCS) is the rate-limiting enzyme of glutathione (GSH) biosynthesis, catalyzing the ATP-dependent ligation of glutamate and cysteine to form γ-glutamylcysteine—the immediate precursor of GSH—and thereby directly dictating the overall rate and capacity of GSH synthesis *in vivo* (Griffith, 1999). Studies have demonstrated that AAEO upregulates the cystine/glutamate antiporter the cystine/glutamate antiporter solute carrier family 7 member 11 (SLC7A11, also known as xCT) (Koppula et al., 2021), thereby augmenting the import of cystine—the rate-limiting precursor for glutathione synthesis. The resultant increase in intracellular cyst(e)ine indirectly activates γ-glutamylcysteine synthetase (γ-GCS), driving *de novo* GSH production and preserving the antioxidant capacity of the glutathione system from its inception, as exemplified by its ability to suppress ferroptotic cell injury (Zhang et al., 2022). Collectively, AAEO orchestrates a multi-target enhancement of GSH recycling, utilisation and biosynthesis, reinforcing antioxidant defence against oxidative stress-related pathologies including chemical-induced hepatotoxicity and organ senescence.

Ultraviolet irradiation can induce the production of ROS in skin tissue, causing oxidative stress and stimulating melanin production (Wood and Schallreuter, 1991). Melanin production can be reduced by antioxidant reducing agents, which interact with copper ions or orthoquinone in the active site of tyrosinase, thereby blocking the oxidative polymerization of melanin intermediates (Karg et al., 1993). As an antioxidant, AAEO can reduce the production of melanin in B16F10 cells, indirectly exerting anti-aging and skin-whitening effects (Sárdy, 2009; Wlaschek et al., 1995; Huang et al., 2012). In addition, AAEO can effectively reduce ethanol-induced liver pathological damage by regulating the composition of intestinal flora, accompanied by increased expression of interleukin-10 (IL-10), and decreased levels of interleukin-1β (IL-1β) and transcript levels of genes encoding components of the peroxisome proliferator-activated receptor γ/nuclear factor κ-light-chain-enhancer of activated B cells (NF-κB) pathway (Wlaschek et al., 1995). This finding suggests that AAEO may alleviate oxidative stress by regulating the expression of inflammation-related cytokines.

### 3.2 Anti-inflammation and immunomodulation effects

Inflammation and immune responses are tightly interconnected, with some researchers coining the term “inflammatory immune responses” to describe the moderate or abnormal systemic reactions of inflammation-related immune cells to internal and external environmental stimuli (Ursini and Maiorino, 2020; Wei, 2016). Immune cells activated by an antigen produce a variety of cytokines, chemokines, and inflammatory mediators [nitric oxide (NO), prostaglandin E2 (PGE2), ROS, tumor necrosis factor-alpha (TNF-α), interferons (IFNs), monocyte chemoattractant protein, and interleukins (ILs)]. The production of these factors leads to intracellular signaling and chemotactic responses in more inflammatory cells, thereby protecting the body from infection (Ramana et al., 2006). Although various cytokines and mediators that promote inflammation play a crucial role in regulating immune responses, their dysregulation can cause damage and lead to various acute and chronic diseases, including cancer, diabetes, septic shock, autoimmune diseases, and atherosclerosis. AAEO participates in the regulation of the dynamic balance of cytokines, inflammatory mediators, and their receptor signaling networks. In this way, it is involved in the control of cells’ pathological responses and the maintenance of normal physiological functions (Choi et al., 2011).

AAEO exerts anti-inflammatory effects through distinct mechanisms in various disease models. In TPA-induced mouse ear edema, AAEO reduces inflammation in a dose-dependent manner, achieving an inhibition rate of 84%, which is comparable to that of indomethacin. This anti-inflammatory effect is mediated by dual regulation of the JAK/STAT and NF-κB signaling pathways: AAEO inhibits the phosphorylation of JAK2 and STAT3, thereby reducing the transcriptional activation of pro-inflammatory factors (NO, PGE2, ROS). Meanwhile, its active component borneol blocks the phosphorylation and degradation of IκBα, preventing the nuclear translocation of the NF-κB p65 subunit. In LPS-stimulated RAW264.7 macrophages, AAEO decreases the nuclear expression of NF-κB p65 and downregulates the mRNA levels of TNF-α and IL-6. Additionally, AAEO inhibits the activation of the MAPK pathway, as evidenced by a reduction in phosphorylated p38 MAPK levels in LPS-induced macrophages (Huang et al., 2024; Tabas and Glass, 2013). In that model, the anti-inflammatory activity of AAEO was attributed to its ability to suppress the Janus kinase/signal transducers and activators of transcription (JAK/STATs) signaling pathway, resulting in decreased production of pro-inflammatory factors (NO, PGE2, ROS) and inflammatory mediators [TNF-α and interleukin-6 (IL-6)] (Tabas and Glass, 2013). In broiler hens infected with infectious bronchitis virus, intranasal administration of AAEO alleviates tracheal mucosal edema and bleeding, reducing mortality by 20.8% (to 4.2% compared with the untreated group). This effect is achieved by downregulating key NF-κB factors and upregulating TLR7 in the MyD88-dependent pathway, thereby enhancing antibody and IFN levels (Chen et al., 2017). The results of real-time quantitative polymerase chain reaction (RT-qPCR) and other experiments showed that AAEO attenuated the inflammatory response mainly by reducing the expression of the key factors in the NF-κB signaling pathway and enhancing the expression of Toll-like receptor 7 (TLR7) in the myeloid differentiation primary response gene 88 (MyD88)-dependent signaling pathway to increase the levels of antibodies and IFNs (Chen et al., 2017).

The role of AAEO in reducing asthma symptoms was explored using the double antibody sandwich method, enzyme-linked immunosorbent assay (ELISA), and other techniques. The results showed that AAEO inhibited 5-lipoxygenase expression to inhibit arachidonic acid-related metabolism of cysteinyl leukotrienes (CysLTs) and increase lipoxin A4 levels. Thus, AAEO reduced the accumulation of CysLTs to alleviate the inflammatory state. Furthermore, AAEO enhanced the activity of indoleamine 2,3-dioxygenase 1 (IDO-1) and modulated the IDO-1-kynurenine (IDO-1-KYN) pathway, thereby upregulating the mRNA expression of interleukin-10 (IL-10) and forkhead box P3 (Foxp3), as well as the transcript level of interleukin-17A (IL-17A). This subsequently downregulated the expression of retinoic acid-related orphan receptor γt (RORγt), which ultimately corrected the imbalance between regulatory T cells (Tregs) and T helper 17 (Th17) cells (Wan et al., 2024).

In the mouse lipopolysaccharide-induced liver injury model, AAEO inhibited the expression of cyclooxygenase-2 (COX-2), NF-κB, and IDO-1, regulated arachidonic acid, purine, and tryptophan metabolism, and enhanced the body’s anti-inflammatory ability (Rong et al., 2024). In addition, AAEO inhibited transient receptor potential A1 channels in sensory nerves, thereby reducing the release of calcitonin gene-related peptide.

**FIGURE 2:**
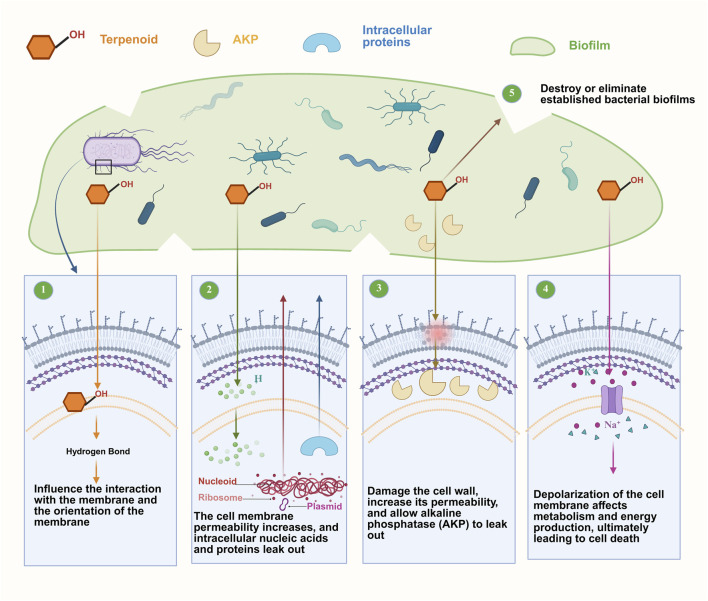
Anti-inflammatory mechanisms of AAEO. Abbreviations: TPA, tissue plasminogen activator; JAK, Janus kinase; STAT, signal transducers and activators of transcription; NO, nitric oxide; PGE2, prostaglandin E2; ROS, reactive oxygen species; TNF-α, tumor necrosis factor-α; TLR7, Toll-like receptor 7; MyD88, myeloid differentiation primary response gene 88; CysLTs, cysteinyl leukotrienes; IDO-1, indoleamine 2,3-dioxygenase 1; CGRP, calcitonin gene-related peptide; RORγt, retinoic acid-related orphan receptor γ t; Foxp3, forkhead box P3; IL-17A, interleukin-17A; L-Trp, L-tryptophan; NF-κB, nuclear factor κ-B; RORγt, retinoic acid receptor-related orphan receptor γt; IκB, inhibitor of NF-κB; IFNs, interferons; dTRPA1, transient receptor potential A1; Treg, regulatory T cells; Th17, T helper 17 cells.

The anti-inflammatory activity of AAEO has also been exploited by using it to treat allergic contact dermatitis (Zhou et al., 2024). The anti-inflammatory activity of AAEO is summarized in Figure 2.

The anti-inflammatory activity of AAEO is closely related to 1, 8-cineole, eugenol, borneol, and other plant metabolites (Yan et al., 2024; César Da Silva et al., 2019). 1, 8-cineole has been demonstrated to stimulate human blood monocytes to produce TNF-α, IL-1β, and thromboxane B2 (TXB2) *in vitro*. TXB2 and PGE2 inhibit the production of inflammatory mediators (Arranz et al., 2014). In addition, 1, 8-cineole has been shown to be effective in reducing paw edema induced by carrageenan, dextran, histamine, and arachidonic acid, as well as exudates induced by the Evans Blue extravasation test. It has an inhibitory effect in experimental inflammation models, such as the carrageenan-induced paw edema and cotton granulomas models in rats (Santos and Rao, 2000). Similarly, the anti-inflammatory mechanism of eugenol may be related to the inhibition of prostaglandin synthesis or the release of other endogenous mediators (Martins et al., 2017). In an experiment on mice, compared with the control group, those supplied with dietary eugenol (50, 75 and 100 mg/kg) had significantly lower scores in an acetate-induced torsion test (61.6%, 64.8%, and 88.3%, respectively), equivalent to the effects of the non-steroidal anti-inflammatory drugs indomethacin and celecoxib (Martins et al., 2017). In addition to inhibiting the release or synthesis of inflammatory mediators, borneol was found to inhibit TNF-α and induce the expression of inducible nitric oxide synthase and IL-1β (Daniel et al., 2009; Zhang et al., 2018). Borneol also effectively treats acne because of its anti-inflammatory and analgesic effects, and was found to inhibit acne-mediated production of IL-6, IL-1β, interleukin-8 (IL-8) and TNF-α by inhibiting the NFκB activation pathway (Zhang et al., 2017).

### 3.3 Anti-pathogenic microorganism effect

The invasion of foreign microorganisms can destroy the original microbial environment of the human body, attack the immune system, and cause damage. These harmful microorganisms are called pathogens. Pathogens can be classified into three groups: acellular organisms (e.g., viruses and prions), prokaryotic organisms (e.g., bacteria, *mycoplasma*, and spirochetes), and eukaryotic organisms (e.g., fungi and protozoa). Traditionally, pathogenic microorganisms are controlled using antibiotics, including cefoperazone, amoxicillin, and cefoxitin. However, long-term improper use of these antibiotics may induce pathogen resistance and result in excessive antibiotic residues in food and the environment, posing risks to human health (Fan et al., 2015; Ding et al., 2023).

Scientists worldwide are searching for safer and more effective anti-pathogen drugs. Since artemisinin became the first choice for the treatment of malaria, there has been increasing interest in the anti-pathogenic microbial mechanism and active ingredients of medicinal plants. Plant essential oils have potential anti-pathogen effects because they contain a wide range of antibacterial and antioxidant components. Plant essential oils are classified as Generally Recognized as Safe (GRAS) by the Food and Drug Administration (FDA) because of their eco-friendly nature. AAEO has a strong inhibitory effect on all the pathogens mentioned above (Wang et al., 2017), and thus it has been widely studied.

When compared with other GRAS-certified essential oils (e.g., *Eucalyptus globulus* essential oil, Zanthoxylum armatum essential oil), AAEO shows distinct advantages and limitations in anti-pathogenic activity. For antibacterial effects: Studies have shown that AAEO exhibits significant antibacterial activity against *Staphylococcus aureus* and *Escherichia coli*. In general, AAEO has been found to be effective against these pathogens, although the exact MIC values may vary depending on the specific source and composition of the essential oil (Liu et al., 2025). In contrast, *E. globulus* essential oil, which contains a high concentration of 1,8-cineole, has been reported to have strong antibacterial activity against *S. aureus* and *E. coli* due to its ability to disrupt bacterial membranes more effectively (Jin, 2025). Additionally, AAEO has a broader spectrum of activity: it has been shown to inhibit *Pseudomonas aeruginosa*, whereas Zanthoxylum armatum essential oil, which is rich in linalool, does not exhibit activity against *P. aeruginosa* (Liang et al., 2025). For antiviral effects: AAEO has been demonstrated to inhibit HBV DNA replication through mechanisms involving the upregulation of TLR7 and activation of the MyD88-IFN pathway. On the other hand, *E. globulus* essential oil has been shown to have antiviral activity by directly binding to the SARS-CoV-2 Mpro active site, blocking viral polyprotein cleavage. No antiviral activity has been reported for Zanthoxylum armatum essential oil (Jin, 2025).

#### 3.3.1 Antibacterial effect

AAEO has antibacterial activity against both Gram-positive bacteria (including *S. aureus*, *Bacillus* cereus, *Bacillus subtilis*, *Listeria*) and Gram-negative bacteria (including *E. coli*, *P. aeruginosa*, *Salmonella typhimurium*) (Liu et al., 2021; Wen et al., 2024).

**FIGURE 3:**
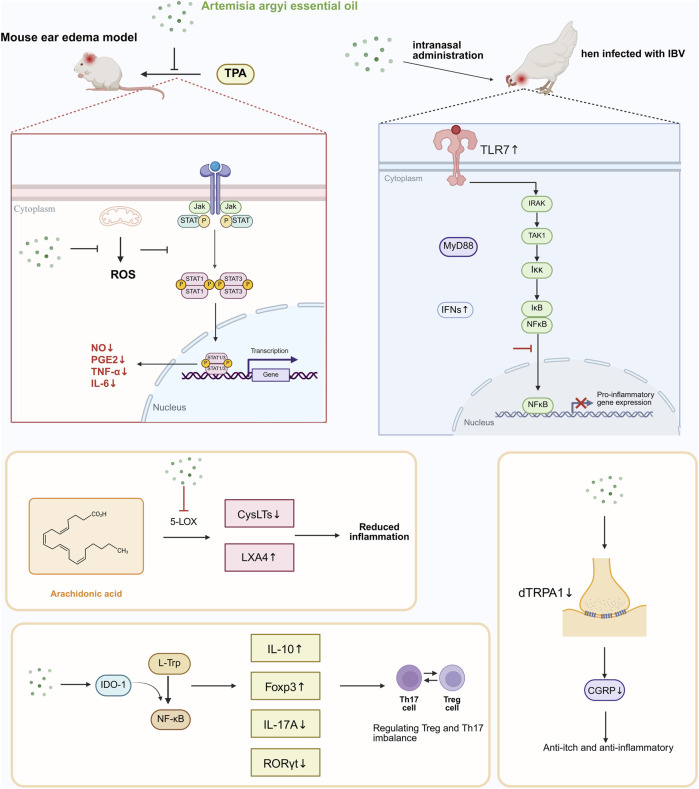
Antibacterial mechanisms of AAEO. Terpenoid plant metabolites of AAEO can degrade biofilm and cell wall of bacteria, and form hydrogen bond with cell membrane, leading to leakage of AKP and nucleic acids. In addition, terpenoid plant metabolites can induce depolarization of cell membrane, leading to disturbance of metabolism and energy production, and finally cause cell death.

Studies have shown that the antibacterial effect of AAEO is due to the synergistic effect of many plant metabolites (Ben Arfa et al., 2006). Several terpenoids — borneol, α-terpineol, terpine-4-ol, 1,8-camphor, caryophyllene oxide, and eugenol — are the key active plant metabolites (Ding et al., 2023). Most of these terpenoids have hydroxyl groups and a certain degree of electron delocalization, and these characteristics are related to their mechanism of action (Wen et al., 2024). Hydroxyl groups allow plant metabolites form hydrogen bonds with bacterial cell membrane phospholipids, which affects the interaction with the membrane and its orientation (Ben Arfa et al., 2006). The electron delocalization of terpenoids gives them the ability to freely diffuse on both sides of the bacterial cell membrane and exchange ions (Ben Arfa et al., 2006). This reduces the resistance of the membrane to protons and large ions (Ben Arfa et al., 2006) and increases membrane permeability (Veldhuizen et al., 2006), leading to the leakage of intracellular nucleic acids and proteins (Moumni et al., 2020). In addition, α-terpinol increases the permeability of the bacterial cell wall, enabling leakage of alkaline phosphatase, which is located between the cell wall and the cell membrane and is involved in many bacterial synthetic and metabolic processes (Moumni et al., 2020). In addition, these terpenoid plant metabolites depolarize the cell membrane potential, affecting metabolism and energy production and ultimately leading to cell death (Huang et al., 2021). Studies have shown that terpine-4-ol can disrupt or even eliminate bacterial biofilms, thereby disrupting the integrity and physiology of bacterial cells (Battistini et al., 2019). The antibacterial mechanism of AAEO is shown in Figure 3.

#### 3.3.2 Antifungal effect

AAEO inhibits a wide range of fungi, including *Candida albicans, Aspergillus Niger, Aspergillus flavus*, and *Metarhizium*, to varying degrees (Kamiya et al., 2024; Zheng et al., 2019). Its antifungal mechanism is similar to its anti-bacterial mechanism, and is attributed to the high lipophilicity of terpene plant metabolites. Treatment with AAEO increases the fluidity and permeability of the fungal cell membrane, which leads to the destruction of fungal cells (Hu et al., 2020; Nakhaee Moghadam et al., 2021).

#### 3.3.3 Antiviral effect

The antiviral effect of AAEO has been studied in several animal and *in vitro* models. In infectious bronchitis virus-infected broiler hens, AAEO treatment reduced mortality. Further analysis revealed that AAEO upregulates the expression of TLR7—a key pattern recognition receptor for avian virus recognition—which activates immune cells via the MyD88-dependent signaling pathway, ultimately increasing the levels of virus-neutralizing antibodies and IFNs (Chen et al., 2017).

AAEO was shown to inhibit the replication of hepatitis B virus’s DNA, and decrease the secretion of the hepatitis B surface antigen and hepatitis B E antigen (Hu et al., 2020). The underlying mechanism involves the regulation of the TLR7-MyD88-IFN signaling pathway: AAEO upregulates the expression of TLR7 in HepG2.2.15 cells (a HBV-expressing cell line), which activates MyD88-dependent signaling, leading to the phosphorylation of IRF7 and NF-κB (Marié et al., 2000). Activated IRF7 promotes the transcription of IFN-α/β, and the increased IFN-α/β further activates the JAK-STAT pathway, upregulating the expression of antiviral proteins such as MxA and OAS1, thereby inhibiting HBV DNA replication. Moreover, AAEO-loaded nanostructured lipid carriers (AAEO-NLC) enhance the inhibition of HBsAg and HBeAg secretion, which is approximately twice the effect of free AAEO, due to improved liver targeting and cellular uptake (Chathuranga et al., 2021; Mukherjee et al., 2015). Studies have shown that AAEO at doses up to 3,000 mg/kg has no obvious toxic effect on mice, suggesting that it may be a potential antiviral drug for the treatment of hepatitis B infection (Zengin and Baysal, 2014).

Analyses of the plant metabolites of AAEO have shown that 1, 8-cineole, camphor, borneol, and α-terpineol have anti-viral effects against a number of viruses. Among those plant metabolites, 1, 8-cineole was identified as the one with the strongest antiviral activity (Huang et al., 2021; Zhang N. et al., 2023; Lai et al., 2017; Yang et al., 2010; Sokolova et al., 2021). Experiments using a herpes simplex virus (HSV) model showed that 1, 8-cineole combines with the glycoproteins gB and gD and the heterodimer gH-gL in the lipid bilayer envelope. This inhibits binding with their receptor, which is associated with the fusion of the virus with the host cell’s plasma membrane, thus blocking the fusion of the lipid bilayer envelope of the virus with host cells (Astani et al., 2010; Minami et al., 2003). Similarly, 1, 8-cineole acts against influenza A virus (H1N1) by binding and inhibiting its hemagglutinin protein. This prevents the virus from binding to its receptor and prevents its subsequent entry into the host cells (Mieres-Castro et al., 2021). Co-administration of 1, 8-cineole (12.5 mg/kg) with influenza virus antigen was shown to provide cross-protection against influenza virus in a mouse model (Li, Y. et al., 2017). For severe acute respiratory syndrome coronavirus 2 (SARS-CoV-2, the causative agent of COVID-19), the main protease (Mpro)—a key homodimeric cysteine protease—cleaves polyproteins into individual proteins essential for viral replication and transcription (Chandorkar et al., 2021; Silva et al., 2020; Fitriani et al., 2020; Abbass, 2020). In silico molecular docking experiments demonstrated that 1,8-cineole binds to amino acids in the Mpro active site and inhibits its activity, supporting its potential application in COVID-19 treatment (Mieres-Castro et al., 2021; Abbass, 2020). The taxonomic validity of *E. globulus* Labill. has been verified via Plants of the World Online (http://www.plantsoftheworldonline.org), and it is recognized as an accepted species in the family Myrtaceae, genus Eucalyptus, serving as a classic source of 1,8-cineole for pharmacological research.

Structural modifications of active plant metabolites can generate new antiviral drugs. Among the plant metabolites of AAEO, camphor and borneol have been subjected to structural modifications for this purpose. For example, Zhao et al. (2015) synthesized plant metabolites 1–3 containing camphor fragments (derived from AAEO plant metabolites) as novel non-adamantane ion channel inhibitors of influenza A virus (Panikar et al., 2021). Kononova et al. (2017) synthesized a series of N-heterocyclic borneol derivatives as inhibitors of viral entry into a human cell line (specifically, entry of Marburg virus). Among them, compound 6 with a methyl piperidine moiety showed the strongest virus-specific activity. Its ability to inhibit Marburg virus entry (ICMarV50 = 4 μM; P < 0.05) was comparable to that of the well-known filovirus infection inhibitor, ion channel inhibitor verapamil (IC50 = 13 µM) (Zhao et al., 2015). Experiments using an *in vitro* system showed that the antiviral effect of α-terpineol on herpes simplex virus type 1 was due to its ability to inhibit viral proliferation (Sokolova et al., 2021) (Table 3).

**TABLE 3:** Antiviral effects of AAEO and its component active plant metabolites.

Active substance	Chemical structure	Types of viruses	Mechanism of Action	References
1,8-Cineole	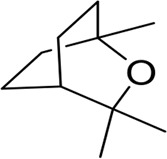	HSV-2	Block the fusion of the lipid bilayer envelope of the virus with host cells	Minami et al., 2003, Mieres-Castro et al., 2021
1,8-Cineole		FM/47/H1N1	Cross protection against influenza virus	Li et al. (2017)
1,8-Cineole		2019-nCoV/SARS-CoV-2	Interaction with Mpro active site	Silva et al. (2020)
AAEO	-	HBV	Regulate inflammation and immune response, and inhibit virus replication	Zhang N. et al. (2023)
AAEO		IBV	Weakening inflammatory response and increasing levels of antibodies and IFN	Wan et al. (2024)
N-heterocyclic borneol derivative 6	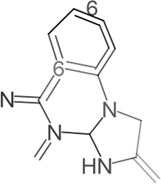	MARV	Specific inhibition of MARV invasion	Kononova et al. (2017)
α-Terpineol	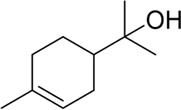	HSV-1	Inhibit virus proliferation	Astani et al. (2010)
Camphor derivatives 1-3	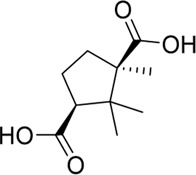	H1N1	M+2 ion channel suppression	Zhao et al. (2015)

### 3.4 Effects of inhibiting cancer cell proliferation and inducing apoptosis

Several studies have explored the effects of AAEO and its constituent plant metabolites on inhibiting cancer cell proliferation and inducing apoptosis. AAEO was shown to induce apoptosis of pancreatic cancer cells (HPAC) by promoting iron imbalance, disrupting the γ-glutamyl cycle, and increasing the content of polyunsaturated fatty acids (Kononova et al., 2017). It was also found to significantly inhibit the migration of liver cancer cells by down-regulating the expression of DEEPDC1 and blocking the Wnt/β-catenin pathway (Zhang Z. L. et al., 2023). In mouse skin melanoma cells (B16F10) stimulated by α-melanocyte stimulating hormone, AAEO inhibited the production of melanin by down-regulating tyrosinase activity and/or reducing oxidative stress (Li B. et al., 2021; Kim and Hong, 2024).

A variety of substances in AAEO have significant potential to inhibit cancer cell proliferation and induce apoptosis. In in vitro analyses, -caryophylene and -caryophylene oxide were able to induce apoptosis and inhibit proliferation of breast cancer cells (MCF-7) and prostate cancer cells (PC-3) (Kim and Hong, 2024), but only detected changes in marker levels, without functional experiments such as angiogenesis tube formation assays to verify the effect on tumor angiogenesis. Both of these plant metabolites reduced the levels of tumor angiogenesis and metastasis markers. The molecular mechanism of their activity of inhibiting cancer cell proliferation and inducing cancer cell apoptosis includes activation of the mitogen-activated protein kinase (MAPK) pathway and inhibition of other pathways [the phosphoinositide 3-kinase (PI3K)/protein kinase B (AKT1)/mechanistic target of rapamycin (mTOR)/S6 kinase 1 (S6K1) and signal transducer and activator of transcription 3 (STAT3) pathways] (Kim and Hong, 2024), However, this study lacks verification using specific pathway inhibitors or gene knockout models, unable to confirm the causal relationship between pathway changes and cell proliferation/apoptosis. 1, 8-cineole was found to inhibit the growth and proliferation of non-small cell lung cancer cells (A549) (Rodenak-Kladniew et al., 2020b) and liver cancer cells (HepG2) (Rodenak-Kladniew, et al., 2020a) by arresting the cell cycle, while the absence of data for key regulators such as Cyclin D1 and p21 leaves the precise molecular basis of the arrest unresolved. It was also shown to increase the expression of COX-2 encoding cyclooxygenase-2 to inhibit ultraviolet B (290–320 nm)-induced skin cancer growth and epidermal thickening, however, the experiment used a mouse skin cancer model induced by ultraviolet radiation, which is different from the pathological process of spontaneous human skin cancer (Lee et al., 2017). The cyclic monoterpene camphene, isolated from the essential oil of *Piper cernuum* Vell. (Piperaceae), has a great transdermal absorption capacity (Obata et al., 1990) and showed lethal effects against subcutaneously transplanted melanoma cells (B16F10-Nex2) after peritumoral administration, the animal model used nude mice with defective immune function, may unable to evaluate the synergistic effect of camphene with the body’s anti-tumor immune response (Girola et al., 2015). The taxonomic validity of *P. cernuum* Vell. has been verified via Plants of the World Online (http://www.plantsoftheworldonline.org), and it is recognized as an accepted species in the family Piperaceae, genus Piper. Further studies suggested that its mechanism involves the condensation of endoplasmic reticulum (Yamaguchi and Wang, 2004; Shiraishi et al., 2006) and activation of the immune system (Kroemer et al., 2013). Thujone showed potent anticancer activity against human cervical squamous carcinoma cells (SiHa). Molecular docking analyses identified its potential targets as mTOR, PI3K, AKT1, B-cell lymphoma-2 (Bcl-2), and mouse double minute 2 (MDM2). These target proteins play an important role in the crosstalk between apoptosis and autophagy (Pal et al., 2022). In a glioblastoma cell system, thujone (isolated from *Thuja occidentalis* L., Cupressaceae) reduced cell viability and exhibited potent anti-proliferative, pro-apoptotic, and anti-angiogenic effects (Torres et al., 2016) (Table 4). Taxonomic validity of *T. occidentalis* L. was confirmed from Plants of the World Online (http://www.plantsoftheworldonline.org). It is accepted in family Cupressaceae, genus Thuja, with good taxonomic validity in medicinal plant research. However, our current evidence is still limited to most experiments were performed in only one cancer cell line or in immunodeficient mice, while the tumor microenvironment and the immune-mediated interaction were not considered. In addition, comparative cytotoxicity of terpenoid plant metaboles on normal cells were not reported, so the complete evaluation of safety before clinical application were lacking.

**TABLE 4:** Core plant species involved in pharmacological experiments: Taxonomic validation summary.

Plant common name	Full scientific name (with authorities)	Family	Taxonomic validation result (plants of the world online)	Core pharmacological role in this review	References
Mugwort	*Artemisia argyi* H. Lév. & Vaniot	Asteraceae	Accepted Name; no synonyms; valid taxonomic status	Core research object; source of AAEO with antioxidant/anti-inflammatory/anticancer/antifungal activities	Liang et al., 2020; Chen C.J. et al., 2021
Cang-ai	*Artemisia argyi* cv. ‘Cangai'	Asteraceae	Accepted cultivar; synonym of *Artemisia argyi*; valid status	Source of CAVO with antidepressant (CUMS model) and neuroprotective (MCAO model) activities	Zhang et al., 2022; Zhang et al., 2021
Capillary Wormwood	*Artemisia capillaris* Thunb	Asteraceae	Accepted Name; type species of Artemisia section Abrotanum	References standard for antifungal activity; its essential oil is used to compare AAEO’s anti-Candida efficacy	Hu et al. (2020), Feng et al. (2021)
Black Pepper relative	*Piper cernuum* Vell	Piperaceae	Accepted Name; valid species in Piper genus	Source of camphene (anticancer metabolite with transdermal absorption capacity)	Girola et al. (2015)
Tasmanian Blue Gum	*Eucalyptus globulus* Labill	Myrtaceae	Accepted Name; type species of Eucalyptus	Source of 1,8-cineole (antiviral/anti-inflammatory metabolite)	Mieres-Castro et al. (2021)
Eastern Arborvitae	*Thuja occidentalis* L	Cupressaceae	Accepted Name; widely recognized medicinal species	Source of thujaplicin derivatives (antioxidant metabolites); referenced for terpenoid biosynthesis	Torres et al., 2016

### 3.5 Other effects

Cang ai volatile oil (CAVO) is a compound volatile oil preparation based on traditional Chinese medicine aromatherapy. The main plant metabolites of CAVO are eugenol, 1, 8-eucalyptol, and terpine-4-ol. Studies have shown that CAVO acts on the central nervous system after nasal inhalation (Chen et al., 2019; Huang et al., 2016). The mechanism of CAVO was investigated in a middle cerebral artery occlusion (MCAO) rat model: CAVO treatment improved neurological function, and results from magnetic resonance imaging (MRI) diffusion tensor imaging and neurological deficit scoring showed it reduced cytotoxic edema in the ischemic area and accelerated nerve fiber bundle recovery during the acute phase of cerebral ischemia-reperfusion, confirming its neuroprotective effect against cerebral ischemia-reperfusion injury (Li et al., 2020). In addition, CAVO was found to induce significant excitation in the right frontopolar cortex, which is closely related to emotional processing and executive function, thus improving the depressed mood of healthy subjects. This result indicated that CAVO has potential applications in the treatment of depression (Wei et al., 2023).

AAEO also has strong insecticidal activity. It has been used as fumigant to poison or repel the mosquito Anopheles angelii (Luo et al., 2022). This insecticidal/repellent activity correlates with the relative contents of cineole, β-caryophyllene, caryophyllene oxide, and phytol; differences in these plant metabolites explain the activity variations of AAEO from different geographical regions (Wei et al., 2023). Specifically, Hubei-sourced AAEO exhibited the strongest larvicidal activity (containing >52% cineole and phytol) (Luo et al., 2022). In terms of a repellent effect, the AAEO from plants growing in Gansu province showed the same high level of protection as N, N-diethyl-3-methyl benzoyl amide (DEET), possibly because this essential oil contained more than 22% eucalyptin (Wei et al., 2023). Terpenoids (eudestin), diterpenoids (phytol), and sesquiterpenoids (caryophyllene) are the main plant metabolites with toxic effects when AAEO is used as a fumigant (Sfara et al., 2009).

AAEO and some of its constituents have analgesic (pain-relieving) activity. Prostaglandins are endogenous active substances that not only cause pain, but also significantly increase the sensitivity of nociceptors to bradykinin and other pain-causing substances, leading to chronic pain (Wang et al., 2017). When mice were gavaged with AAEO at different doses, 0.5%–2.0% AAEO significantly reduced the content of prostaglandin E2 in the blood. The ability of AAEO to reduce the expression of pain-causing substances explained its analgesic effect (Wang et al., 2017). The analgesic effect of AAEO may be attributed to active components such as eugenol, borneol and β-caryophyllene. In a mouse model, the group supplied with eugenol (50, 75 and 100 mg/kg) showed significantly reduced acetic acid-induced writhing (61.6%, 64.8%, and 88.3%, respectively, of that in the control group) (Martins et al., 2017). Borneol is used in pharmaceutical preparations for acute pain; its mechanism involves enhancing γ-aminobutyric acid type A (GABAA) receptor-mediated γ-aminobutyric acid (GABA) expression in the spinal cord, improving mechanical hyperalgesia and thus alleviating chronic pain (Jiang et al., 2015). β-caryophyllene relieves acute/chronic pain in mice by activating peripheral cannabinoid receptor 2 (CB2) or the endogenous opioid analgesic system, thereby reducing paclitaxel-induced peripheral neuropathic pain (Segat et al., 2017).

## 4 Conclusion and future perspectives

With the development of safe and effective plant medicines, there has been increasing interest in AAEO in recent years. AAEO has proven beneficial properties and effects, including antioxidant activity, anti-inflammation, immune regulation, and anti-cancer properties, and inhibitory activity against pathogenic microorganisms and mosquitos (the “activity of inhibiting cancer cell proliferation and inducing cancer cell apoptosis” is based on preclinical studies, not yet confirmed by clinical trials). These pharmacological activities are more or less correlated with each other. As mentioned above, AAEO can alleviate inflammation by reducing oxidative stress, thereby contributing to homeostasis of the immune system. All of these pharmacological effects (especially the activity of inhibiting cancer cell proliferation and inducing cancer cell apoptosis) provide a strong basis for the development and utilization of AAEO.

Future research on the development of new plant drugs should build on knowledge about existing drugs. The fact that the anti-inflammatory activity of AAEO is equivalent to that of indomethacin (Tabas and Glass, 2013) should prompt further research into its mechanism of action; that is, whether its pharmacological effects are similar to those of non-steroidal anti-inflammatory drugs (i.e., analgesic and antipyretic functions and the ability to inhibit platelet aggregation). The effective plant metabolites (e.g., 1,8-cineole, β-caryophyllene) can be used as lead compounds for drug development, and new drugs with stronger efficacy and fewer adverse reactions can be designed by modifying their chemical skeletons or adjusting functional groups (e.g., introducing hydroxyl groups to enhance water solubility of thujone) (Pal et al., 2022).

To address AAEO’s intrinsic physicochemical limitations (high lipophilicity, poor water solubility, rapid volatilization, and fast *in vivo* metabolism), advanced nano-carrier systems are critical for its clinical translation, with preclinical studies confirming their efficacy: HP-β-cyclodextrin (HP-β-CD) inclusion complexes: Recent studies have shown that HP-β-CD can significantly enhance the solubility and oral bioavailability of various compounds. For instance, the solubility of altrenogest was increased by 1,026.51-fold through complexation with HP-β-CD, and its oral bioavailability was also improved. Similarly, AAEO-HP-β-CD complexes have been shown to enhance the solubility and stability of AAEO, making it more suitable for oral formulations and reducing gastrointestinal degradation (Chen et al., 2024; Yan et al., 2024). Zhang Z. L. et al. (2023) developed AAEO-NLC with a particle size of 120–150 nm and an encapsulation efficiency of approximately 85%. In HepG2.2.15 cells (HBV-expressing hepatocytes), AAEO-NLC significantly suppressed HBsAg and HBeAg secretion, with reductions of 58.7% and 62.3%, respectively, which is nearly double the activity of free AAEO (31.2% and 35.6%). In a mouse model of HBV infection, AAEO-NLC reduced liver HBV-DNA copies by 1.8 log10 and alleviated hepatic histopathological damage (e.g., reduced hepatocyte necrosis) by targeting the liver via the reticuloendothelial system (Pan et al., 2025). Although AAEO-specific liposomal data are limited, insights from its major component 1,8-cineole support this approach. Recent studies have shown that liposomal formulations of 1,8-cineole can significantly improve its pharmacokinetic properties and therapeutic efficacy. For example, liposomal 1,8-cineole has been shown to extend the half-life from 1.2 h to 4.5 h, which is critical for reducing systemic clearance and improving lung targeting—essential for respiratory antiviral applications (Pries et al., 2023).

To elucidate AAEO’s mechanisms of action, transcriptomics and metabolomics approaches have been employed. RNA-seq analysis of AAEO-treated HepG2 cells revealed differentially expressed genes (DEGs) with enrichment in the Wnt/β-catenin and MAPK pathways. Downregulation of DEPDC1 was confirmed to block Wnt/β-catenin-mediated liver cancer cell migration (Li Y. L. et al., 2021). In LPS-induced RAW264.7 macrophages, AAEO altered the expression of inflammation-related genes, including upregulation of TLR7 and downregulation of NF-κB p65 (Qin et al., 2022). Ultra-performance liquid chromatography-tandem mass spectrometry (UPLC-MS/MS) of AAEO-treated asthmatic mice showed changes in metabolites, particularly in the 5-LOX-CysLTs and IDO-1-KYN pathways. AAEO increased lipoxin A4 levels and decreased kynurenine (KYN), correcting Treg/Th17 cell imbalance. For antioxidant effects, metabolomics identified an increased GSH/GSSG ratio and decreased malondialdehyde (MDA) via regulation of γ-glutamyl cycle enzymes (Rong et al., 2024). Lu et al. constructed a “AAEO-component-target-disease” network, identifying 12 core targets (e.g., mTOR, PI3K, AKT1) for anticancer/antiviral effects. Molecular docking confirmed 1,8-cineole binds to the active site of SARS-CoV-2 Mpro (binding energy: -7.2 kcal/mol) and inhibits its protease activity (Lu et al., 2022).

Before clinical application, comprehensive toxicological evaluations are essential to define safe dose ranges and avoid adverse effects. For acute toxicity, Zengin and Baysal (2014) reported no mortality or obvious organ damage in mice after oral administration of AAEO up to 3,000 mg/kg, with a median lethal dose (LD50) >5,000 mg/kg (Prasanth et al., 2015). Subacute toxicity studies (14-day oral administration of 500, 1,000, 2000 mg/kg AAEO) showed no significant changes in serum alanine transaminase (ALT) or creatinine levels, indicating no hepatotoxicity or nephrotoxicity (Kma et al., 2023). For chronic toxicity, a 90-day study in rats (oral 100, 300, 500 mg/kg AAEO) revealed mild hepatic steatosis only at the highest dose (500 mg/kg), which was reversible after 30 days of drug withdrawal. No changes in hematological parameters (e.g., white blood cell count, hemoglobin) or reproductive toxicity (e.g., sperm motility, follicle count) were observed (Guo et al., 2025). For skin and mucosal irritation, in a rabbit skin irritation test, 2% AAEO cream caused no erythema or edema, meeting the “non-irritating” standard. For nasal administration (used in respiratory studies), AAEO nasal drops (0.5%, 1%) showed no damage to nasal cilia in rats, confirming mucosal safety (Jung et al., 2022).

Many factors affect the quality of AAEO, and its composition and content vary greatly among plants grown in different regions. Therefore, scientific quality control standards should urgently be established. Finally, clinical trials should be conducted to provide clinical data supporting the effectiveness of AAEO. Ongoing trials related to the genus Artemisia, such as NCT05318157, provide valuable insights. This particular trial focuses on specific immunotherapy using Artemisia pollen, enrolling 150 patients with allergic rhinitis. Although the intervention is pollen extract, the safety data generated, including IgE monitoring and adverse reaction rates, can serve as supplementary references for assessing the clinical sensitization potential of AAEO. Additionally, from closely related species can provide considerable support to promising in AAEO. Clinical study has revealed acceptable efficacy and safety of 5% lotion of Artemisia sieberi essential oil usage for treatment of dermatophytosis. These findings offer reference evidence for the potential clinical value of AAEO, thereby reinforcing the assessment of its potential (Stella et al., 2017). In the design of clinical trials, one needs to consider different aspects that would ensure that the trials are carried out in a scientific manner and yield reliable clinical data to support efficacy and safety of AAEO.

## Glossary

AAEO: Artemisia argyi H. Lév. & Vaniot essential oil
ABTS: 2,2′-azino-bis (3-ethylbenzthiazoline-6-sulphonic acid)
AKP: alkaline phosphatase
AKT1: protein kinase B
A549: non-small cell lung cancer cells
B16F10: skin melanoma cells
B16F10-Nex2: melanoma cells
Bcl-2: B-cell lymphoma-2
CAVO: Cang ai volatile oil
CAT: catalase
CB2: cannabinoid receptor 2
COVID-19 or SARS-CoV-2: Coronavirus disease 2019
COX-2: cyclooxygenase-2
CysLTs: cysteinyl leukotrienes
DEET, N: N-diethyl-3-methyl benzoyl amide
DEPDC1: DEP -domain protein containing 1
DPPH: 1,1-diphenyl-2-picrylhydrazyl radical 2,2-diphenyl-1-(2,4,6-trinitrophenyl) hydrazyl
ELISA: enzyme-linked immunosorbent assay
FDA: Food and Drug Administration
GRAS: Generally Recognized as Safe
H1N1: influenza A virus
Hacat: human keratinocytes cells
HBV: hepatitis B virus
HepG2: liver cancer cells
HPAC: pancreatic cancer cells
HSV: herpes simplex virus
HSV-1: herpes simplex virus type 1
HSV-2: herpes simplex virus type 2
IBV: infectious bronchitis virus
IDO-1: indoleamine 2,3-dioxygenase 1
IFNs: interferons
IL-1: interleukin-1
IL-6: interleukin-6
IL-8: interleukin-8
IL-10: interleukin-10
ILs: interleukins
JAK/STATs: Janus kinase/signal transducers and activators of transcription signaling pathway
Keap1: Kelch-Like Epichlorohydrin-associated protein
KYN: kynurenine
MAPK: mitogen-activated protein kinase pathway
MARV: Marburg virus
MCF-7: breast cancer cells
MCP: monocyte chemoattractant protein
MDA: malondialdehyde
MDM2: mouse double minute 2
mTOR: mechanistic target of rapamycin
MyD88: myeloid differentiation primary response gene 88
NF-κB: peroxisome proliferator-activated receptor γ/nuclear factor -light-chain-enhancer of activated B cells pathway
NO: nitric oxide
Nrf2: Nuclear factor erythroid 2-related factor 2
PC-3: prostate cancer cells
PCOs: protein carbonyls
PGE2: prostaglandin E2
PI3K: phosphoinositide 3-kinase
ROS: reactive oxygen species
RT-qPCR: real-time quantitative polymerase chain reaction
S6K1: S6 kinase 1
SiHa: cervical squamous carcinoma cells
SOD: superoxide dismutase
STAT3: activator of transcription 3
TLR7: Toll-like receptor 7
TNF-α: tumor necrosis factor-alpha
TPA: tissue plasminogen activator
TXB2: thromboxane B2
NLRP3: NLR family pyrin domain-containing 3
SLC7A11: solute carrier family 7 member 11 (xCT)
MCAO: middle cerebral artery occlusion
HP-β-CD: hydroxypropyl-β-cyclodextrin
NLC: nanostructured lipid carriers
